# Fifty years of impact on liver pathology: a history of the Gnomes

**DOI:** 10.1007/s00428-020-02879-5

**Published:** 2020-06-30

**Authors:** Michael Torbenson, Valeer Desmet, Helmut Denk, Francesco Callea, Alastair D. Burt, Stefan G. Hübscher, Luigi Terracciano, Hans-Peter Dienes, Zachary D. Goodman, Pierre Bedossa, Ian R. Wanless, Eve A. Roberts, Elizabeth M. Brunt, Andrew D. Clouston, Annette S.H. Gouw, David Kleiner, Peter Schirmacher, Dina Tiniakos

**Affiliations:** 1 Department of Laboratory Medicine and Pathology, Mayo Clinic, Rochester, MN USA; 2 Histology and Pathology, Faculty of Medicine, K.U. Leuven, Leuven, Belgium; 3 Institute of Pathology, Medical University of Graz, Graz, Austria; 4 Ospedale Pediatrico Bambino Gesu, 00165 Rome, Italy; 5 Translational & Clinical Research Institute, Faculty of Medical Sciences, Newcastle University, Framlington Place, Newcastle upon Tyne, NE2 4HH UK; 6 Faculty of Health and Medical Sciences, University of Adelaide School of Medicine, Adelaide, South Australia 5005 Australia; 7 Institute of Immunology and Immunotherapy, University of Birmingham, Birmingham, UK; 8 Department of Cellular Pathology, Queen Elizabeth Hospital Birmingham, Birmingham, B15 2WB UK; 9 Institute of Pathology, University of Basel, 4003 Basel, Switzerland; 10 Institute of Pathology, Meduniwien, Medical University of Vienna, 1090 Wien, Austria; 11 Center for Liver Diseases, Inova Fairfax Hospital, Falls Church, VA 22042 USA; 12 LiverPat, Paris, France; 13 Department of Pathology, Dalhousie University, Queen Elizabeth II Health Sciences Centre, Halifax, Nova Scotia B3H 1V8 Canada; 14 Division of Gastroenterology, Hepatology and Nutrition, The Hospital for Sick Children, Toronto, Ontario M5G1X8 Canada; 15 Department of Pathology and Immunology, Washington University School of Medicine, St. Louis, MO 63110 USA; 16 Centre for Liver Disease Research, School of Medicine (Southern), University of Queensland, Princess Alexandra Hospital, Ipswich Rd, Woolloongabba, 4109 Australia; 17 Department of Pathology and Medical Biology, University Medical Center Groningen, 9700 RB Groningen, The Netherlands; 18 National Institutes of Health, Bethesda, MD USA; 19 Heidelberg University, Im Neuenheimer Feld 224, 69120 Heidelberg, Germany; 20 Department of Pathology, Aretaieion Hospital, Medical School, National & Kapodistrian University of Athens, Athens, Greece

**Keywords:** Liver, Pathology, Scientific group, Model, History

## Abstract

Professional societies play a major role in medicine and science. The societies tend to be large with well-developed administrative structures. An additional model, however, is based on small groups of experts who meet regularly in an egalitarian model in order to discuss disease-specific scientific and medical problems. In order to illustrate the effectiveness of this model, the history and practices are examined of a long-standing successful example, the International Liver Pathology Group, better known as the *Gnomes*. The history shows that groups such as the Gnomes offer a number of important benefits not available in larger societies and nurturing such groups advances science and medicine in meaningful ways. The success of the Gnomes’ approach provides a road map for future small scientific groups.

**Electronic supplementary material:**

The online version of this article (10.1007/s00428-020-02879-5) contains supplementary material, which is available to authorized users.

## Introduction

Professional societies play a major role in shaping concepts, prioritizing academic pursuits, and providing expert guidance for clinical management challenges in patient care. Professional societies in most settings tend to be large in order to maximize impact. They have permanent bureaucratic structures to help them operate consistently and effectively, including official bylaws and standard operating procedures that outline committee structure and committee interactions, with a President and Executive Committee, and frequently a professional manager overseeing the entire operation.

An additional model, however, is based on small groups of experts who meet regularly in an egalitarian model without formal structure in order to discuss disease-specific scientific and medical problems. Of course, this model is not intended to replace or compete with the roles of traditional professional societies, but this model is nimble and flexible and can have a great impact on medicine and science. In order to illustrate this model, the history of a notable example is studied: the International Liver Pathology Group that in 2018 celebrated its 50th anniversary.

## Origin and development of the Gnomes

The International Liver Pathology Group, better known as the *Gnomes*, emerged spontaneously in 1967 when a group of expert hepatologists and liver pathologists met to take on one of the most pressing problems of the day—how should chronic hepatitis be conceptualized, and what terms should be used to capture the various injury patterns seen on liver biopsy? This problem emerged because of the rapid spread during the 1950s and 1960s of the then new technique of using needle biopsies for the diagnosis and management of patients with liver disease. It was clear to pathologists and hepatologists that there were different patterns of hepatic injury, but the different patterns and their significance were confusing, at one point leading to at least 40 different terms in use for chronic hepatitis in the medical literature [1].

This issue was tackled in 1966 by the leadership of the newly formed European Association for the Study of the Liver (EASL) at the first annual meeting in Marburg, Germany. The EASL President, Gustav-Adolf Martini, and the Secretary, Jan De Groote (hepatologist and one of the founding members of the Gnomes from Leuven, Belgium), recognized the need for consensus on terminology for hepatitis injury patterns, so that uniform terminology would be used in the literature and permit fuller and more rapid progress in understanding inflammation of the liver [2]. To address this issue, it was decided to organize a session on this topic for the 2nd annual meeting of EASL, which was to be held in Gothenburg, Sweden, in 1967. Two German pathologists, Peter Gedigk from Bonn and Gerhard Korb from Weiden, were asked to host a slide seminar on this topic; both became founding members of the Gnomes [2]. Hans Popper, an Austrian-born liver pathologist working in New York, USA, had also been invited, but was unable to attend [2]. The session was attended by at least fifty individuals interested in hepatitis. The session was intense, stimulating, and so rewarding that at the suggestion of Jan De Groote a dozen attendees decided to skip the rest of the EASL meeting and continue their discussion on the terminology of hepatitis [3, 4]. They realized they would need more time than this single meeting afforded, so they decided to take their preliminary consensus classification of hepatitis terminology and apply it to a group of circulated slides, and then to meet again. This next meeting, which took place on July 3–5, 1968, at the University of Zürich, Switzerland, is generally considered to be the first meeting and the official birth of the Gnomes, though at this point the group was known as the “European Liver Pathology Group” and had not acquired the moniker of Gnomes.

The Zürich meeting was hosted by Martin Schmid, another founding member of the Gnomes, and was sponsored in part by Hoffman-La Roche & Company [5]. The paper that resulted from the Zürich meeting is titled “A classification of chronic hepatitis” and summarized the new consensus classification of hepatitis developed by the nascent group soon to become known as the Gnomes. At the end of each day of this first meeting, Peter Scheuer (one of the founding members) typed up a summary of the day’s discussion on a borrowed typewriter. As the only fluent English speaker, he became the *de facto scribe* and the meeting summaries formed the basis for the group’s first paper [5]. The paper was quickly published in *The Lancet* in the fall of 1968. The classification system proposed in the paper attempted to identify patterns of hepatitis that were more likely to progress to cirrhosis (chronic aggressive hepatitis) versus those that were thought to be more indolent (chronic persistent hepatitis). This classification system built upon the earlier work of Valeer Desmet [3], which in turn was built on the 1966 publication of Martin Schmid [6], was an important early step in understanding chronic hepatitis and helped lay the foundation for modern understandings of inflammatory liver diseases. The basic dichotomy of chronic aggressive hepatitis versus chronic persistent hepatitis is no longer in use, but the core notion of the importance of “piecemeal necrosis” (now called interface activity) remains relevant to this day.

Following the Zürich meeting, this consensus paper was presented at the 1968 World Congress of Gastroenterology in Prague and subsequently at Karlovy Vary, Czech Republic, for the 1968 meeting of the International Association for the Study of the Liver (IASL) [3, 4]. The president of IASL, Dame Sheila Sherlock, listened with interest but commented later at the meeting banquet, with some disapproval, that the authors were like the “Gnomes of Zürich,” exercising undue influence on the field of liver disease and on the histological terminology of hepatitis [4].

The term *Gnomes of Zürich* referred to a popular notion that a small secretive group of elect bankers in Zürich had an undue influence on the world’s financial systems. When the term *Gnomes* was first used by Sheila Sherlock, it was thus intended to be disparaging. The term, however, was gradually adopted by the group, with a bit of humor and as a badge of honor, being more convenient than the official name European Liver Pathology Group (1968–1978) or the more expansive name International Liver Pathology Group, which was adopted in 1979 when Kamal Ishak joined the group as the first non-European. In publications, the authorship byline was often listed simply as “International Group.” While the more formal name International Liver Pathology Group is still occasionally used, by and large members refer to the group as the Gnomes. Other key events in the history of the Gnomes are listed in Table 1.

**Table 1:** Key events in Gnomes’ history

Date	Key event
1967	Spark that led to the creation of the Gnomes: EASL meeting in Gothenburg, Sweden
1968	First formal meeting of Gnomes: Zürich, Switzerland, July 3–5
	First paper written by Gnomes; drafted at the end of the first meeting by Peter Scheuer; published a few months later (September 1968) in *Lancet*
	The first paper’s classification of hepatitis is presented at the IASL. This leads the IASL president, Dame Sheila Sherlock, to complain the group is acting like the Gnomes of Zürich, who were considered to have undue influence on financial markets
1968	First new member added to the group (Leonardo Bianchi, Basel, Switzerland)
	Formal name for the group is “European Liver Pathology Group” [4]
1976	First woman Gnome: Amelia Baptista
1979	First non-European member added to the group (Kamal Ishak). Hans Popper had joined in 1970 but was largely considered by the group to be European [4]
1983	First meeting held outside of Europe/UK; held in Washington, DC, hosted by Kamal Ishak Formal name is changed to “International Liver Pathology Group” [4]
1989	London meeting inspires Dr Amar Paul Dhillon, a liver pathologist working with Peter Scheuer at the Royal Free Hospital, and two visiting pathologists, Drs Neil Theise and Romano Colombari, to begin a similar group of liver devotees, called the Elves [7]
1990	The official red Gnomes hat was created by the mother of a medical student who was working with Peter Scheuer at the time [2, 7]. The student persuaded his mother to make a hat suitable for a master gnome
2012	First meeting and Liver Symposium in Africa, held in Mwanza, Tanzania, hosted by Francesco Callea
2013	First meeting in Australia, held in Noosa, hosted by Andrew Clouston
2018	50th anniversary meeting held in Athens, Greece, hosted by Dina Tiniakos

## Members

**Fig. 1:**
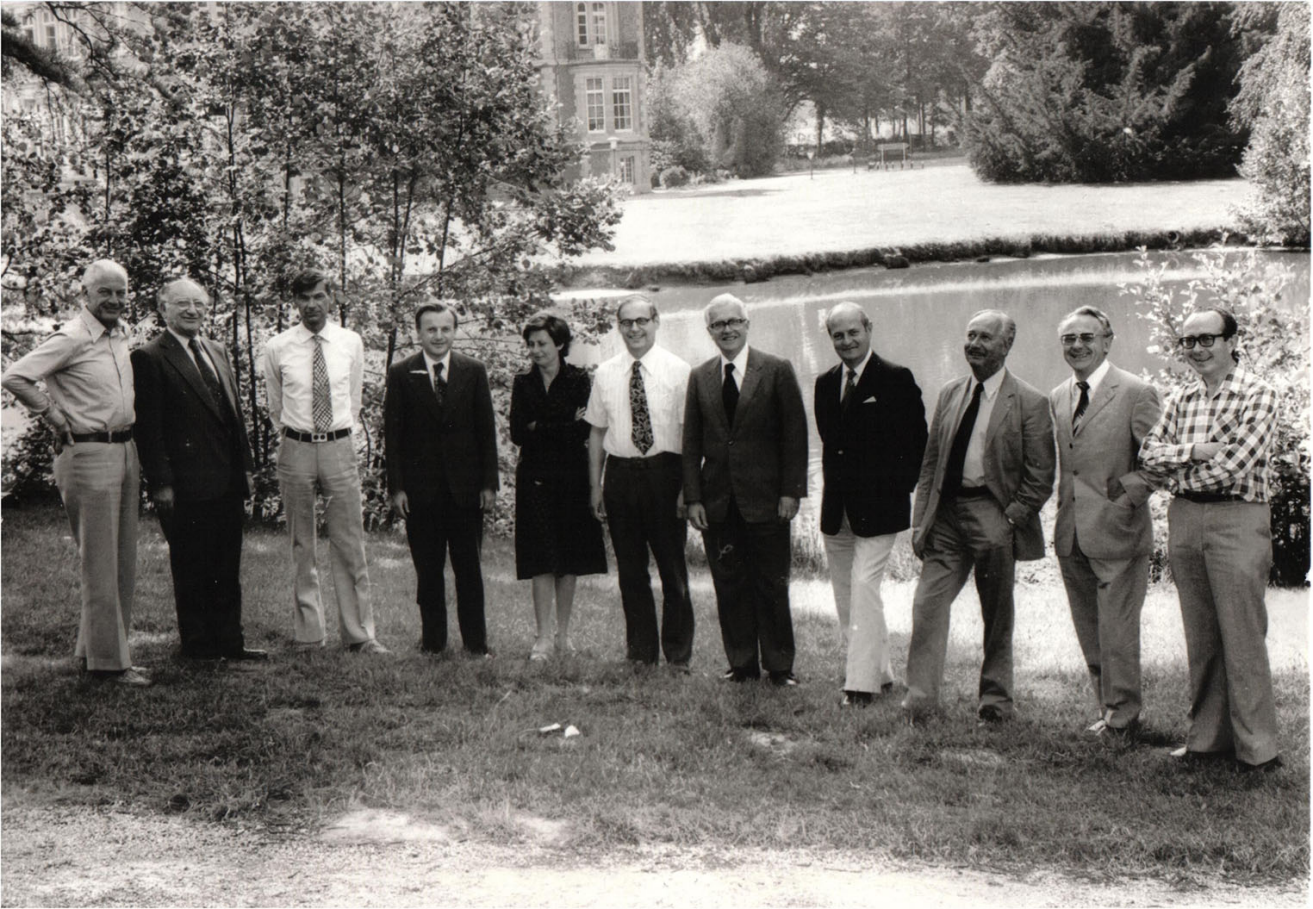
Leuven 1976 meeting—hosts: Jan De Groote and Valeer Desmet. Park of the University Hospital Pellenberg. Left to right: Gnomes Poulsen, Popper, Bianchi, De Groote, Baptista (new member), Scheuer, Gedigk, Schmid, Thaler, Korb, and Desmet

**Fig. 2:**
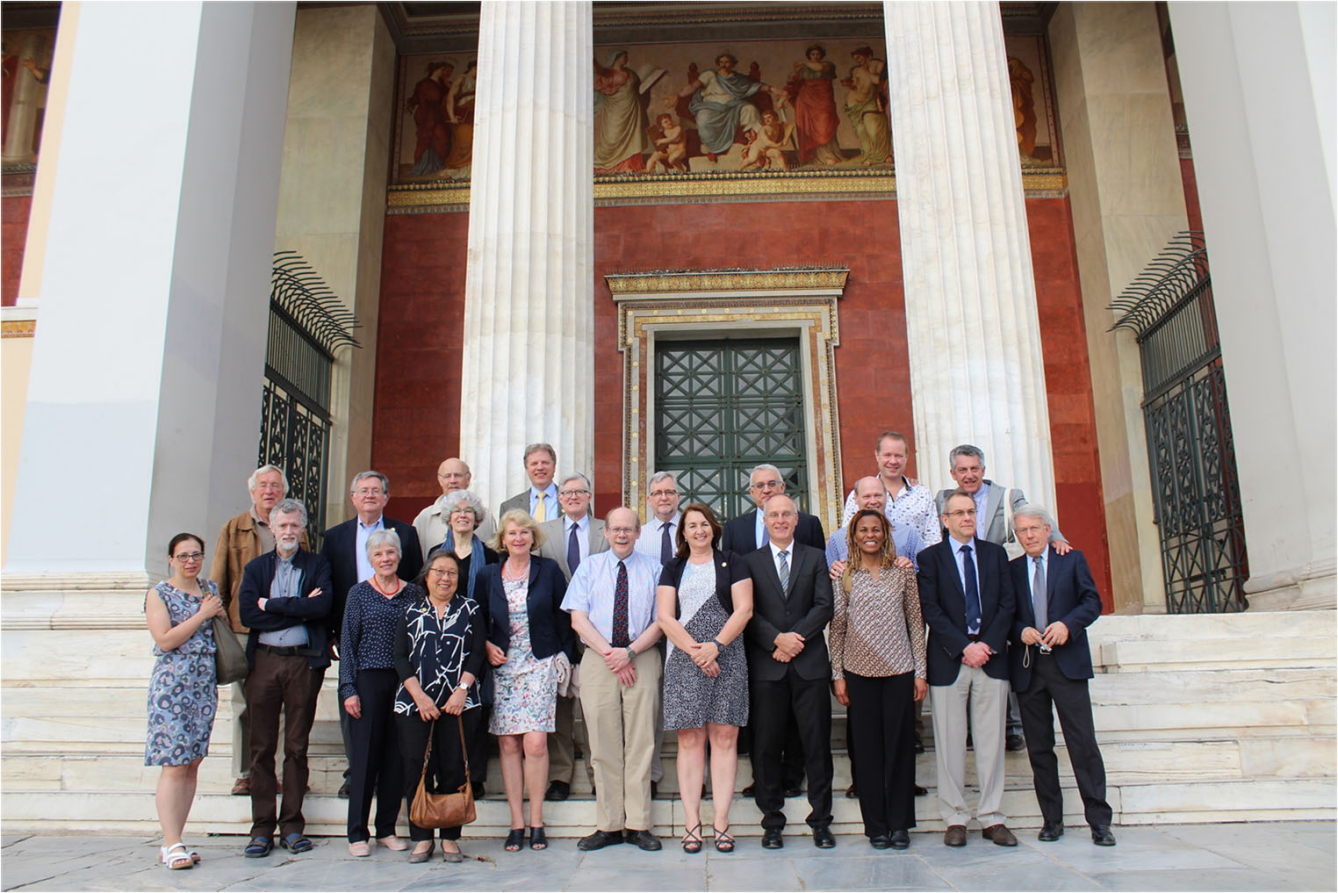
Athens 2018 50th anniversary meeting—host Dina Tiniakos. Back row, left to right: Hans Dienes, Zack Goodman, Pierre Bedossa, Eve Roberts, Mike Torbenson, Ian Wanless, Alastair Burt, Luigi Terracciano, Andrew Clouston, Jim McGown (Gnome mate), Dimitrios Dougenis (Gnome mate). Front row, left to right: Yvonne Bury (observer), Ton Groothuis (Gnome mate), Jamie Goodman (Gnome mate), Annette Gouw, Heidi Dienes (Gnome mate), David Kleiner (new member), Dina Tiniakos, Peter Schirmacher (new member), Vanessa Torbenson (Gnome mate), Stefan Hübscher, and Francesco Callea (Elisabeth Brunt not in attendance)

There were nine founding members of the Gnomes (Table 2). Three of the founding members (De Groote, Thaler, and Schmid) were hepatologists and the rest were pathologists [5]. The group was expanded over the next several years to 11 members and (Fig. 1) held steady at between 11 and 14 members (Fig. 2). This number was felt to be optimal, providing enough members to capture a wide breadth of expertise, but numbers that could still easily meet in a modest-sized room and allow all members to speak freely and informally [8]. New members are inducted when members are no longer able to circulate slides [2] because of retirement, health considerations, scheduling issues, etc. These “*emeriti*” Gnomes are always welcome to continue attending the meeting and otherwise fully participate.

**Table 2:** Gnomes members listed by first year of membership, and city and country of origin. Members who joined the same year are listed in alphabetical order

	Year	Member	City, country
1	1967	Jan De Groote, founding member	Leuven, Belgium
2	1967	Valeer Desmet, founding member	Leuven, Belgium
3	1967	Peter Gedigk, founding member	Bonn, Germany
4	1967	Gerhard Korb, founding member	Weiden, Germany
5	1967	Hemming Poulsen, founding member	Copenhagen, Denmark
6	1967	Peter Scheuer, founding member	London, UK
7	1967	Martin Schmid, founding member	Zürich, Switzerland
8	1967	Heribert Thaler, founding member	Vienna, Austria
9	1967	Wilhelm Wepler, founding member	Kassel, Germany
10	1968	Leonardo Bianchi	Basel, Switzerland
11	1969/70	Hans Popper	New York, NY, USA
12	1976	Amelia Baptista	Lisbon, Portugal
13	1977	Roderick MacSween	Glasgow, UK
14	1979	Kamal Ishak	Washington, DC, USA
15	1986	M. James Phillips	Toronto, Canada
16	1987	Helmut Denk	Graz, Austria
17	1987	Fred Gudat	Basel, Switzerland
18	1992	Francesco Callea	Rome, Italy
19	1992	Bernard Portmann	London, UK
20	1995	Alastair Burt	Newcastle upon Tyne, UK
21	1995	Stefan Hübscher	Birmingham, UK
22	1996	Tania Roskams	Leuven, Belgium
23	1998	Luigi Terracciano	Basel, Switzerland
24	2002	Hans-Peter Dienes	Cologne, Germany
25	2002	Jean-Yves Scoazec	Lyon, France
26	2003	Pierre Bedossa	Paris, France
27	2003	Zachary Goodman	Washington, DC, USA
28	2004	Elizabeth Brunt	St. Louis, MO, USA
29	2004	Eve Roberts	Toronto, Canada
30	2004	Ian Wanless	Toronto, Canada
31	2008	Andrew Clouston	Brisbane, Australia
32	2010	Dina Tiniakos	Athens, Greece
33	2011	Annette Gouw	Groningen, The Netherlands
34	2011	Michael Torbenson	Baltimore, MD, USA
35	2018	David Kleiner	Washington, DC, USA
36	2018	Peter Schirmacher	Heidelberg, Germany

To date, there have been 36 Gnomes. All members were European until Kamal Ishak was inducted into membership in 1979 (Table 2). Technically, Hans Popper had joined in 1970 as an American, but he was considered to be European, and not American, by the members of the Gnomes [4]. The first Canadian joined in 1986 (James Phillips) and the first Australian in 2008 (Andrew Clouston). The first woman Gnome was Amelia Baptista from Lisbon, Portugal, who joined the Gnomes in 1976 (Fig. 1).

When new members are needed, names of candidates are proposed by any active member and discussed by the entire group, with a final decision put to a vote. Criteria for membership are equally weighted towards scientific interest, diagnostic or clinical expertise, collegiality, and friendship [4]. Nonetheless, all candidates are anticipated to be academic leaders in the field of liver disease/liver pathology. For example, the 14 members attending the 2018 meeting in Athens, Greece—the 50th year anniversary meeting (Fig. 2)—had an average *h*-index of 54 (range 27–77). At the time of the 2018 meeting, their individual publications had been referenced in the peer-reviewed literature a total of 202, 550 times, with an average of 14, 468 citations per member (data from World Wide Web of Science, accessed February 4, 2018). In addition, at the time of the 50th year anniversary meeting, they were editors or sole authors on thirteen books on liver pathology, including the seminal *MacSween’s Pathology of the Liver* and the volume on *Liver Tumors* published by the AFIP [9–21]. Finally, current Gnomes members have been or are President or Executive Officers in all of the major International, European, US, Canadian, and Australian professional societies dedicated to pathology and liver pathology, as well as broader liver-focused groups such as the Canadian Association for the Study of the Liver (CASL) and the Austrian Academy of Sciences.

Over the years, the Gnomes have become aware of the critical shortage of pathologists in developing countries. The 2012 meeting, hosted by Franesco Callea, was held in Tanzania to support his ongoing efforts to provide pathology training at the Catholic University of Health and Allied Sciences – Bugando, Mwanza [22].

## Gnomes’ modus operandi

The group originally considered becoming a subcommittee of the EASL, but the members felt there were significant benefits to remaining an independent organization [4]. The group also decided to adopt an egalitarian structure, with no president, no secretary, and no bylaws [4]. Nonetheless, general customs and norms developed over the years for running the meetings (Supplementary material - Appendix). The meeting locations are chosen by the host, often being in the city of the host’s hospital or academic department, and also at nearby resorts. The topics have varied, with most topics focused on medical liver diseases (Table 3). The topics have been wide ranging, covering almost all aspects of medical and tumor liver pathology.

**Table 3:** Calendar of Gnomes’ meetings

Year	Meeting Location	Host	Topic
1967	Gothenburg Sweden	EASL	Classification of chronic hepatitis
1968	Zürich, Switzerland	Martin Schmid	Classification of chronic hepatitis
1969	Leuven, Belgium	Jan De Groote Valeer Desmet	Acute versus chronic hepatitis markers of chronicity
1970	Copenhagen, Denmark	Hemming Poulsen	Evolution of acute to chronic hepatitis
1971	London, UK	Peter Scheuer	Drug-induced and toxic liver damage
1972	Kassel, Germany	Wilhelm Wepler	Cholestatic versus hepatic liver disease
1973	Macerata, Italy *Invitation of Prof. Giorgio Menghini*	Gerhard Korb	Drug-induced hepatitis and cholestasis
1974	Basel, Switzerland	Leonardo Bianchi	Outcomes of forms of chronic hepatitis (chronic persistent and aggressive hepatitis)
1975	Bonn, Germany	Peter Gedigk	Aspects of severity of chronic hepatitis: confluent (bridging) necrosis and its evolution
1976	Leuven, Belgium	Jan De Groote Valeer Desmet	Definition and importance of confluent necrosis and of piecemeal necrosis
1977	Vienna, Austria	Heribert Thaler	Alcoholic liver disease
1978	Zürich, Switzerland	Martin Schmid	Forms of alcoholic liver disease (cholestatic; rapidly evolving, etc.) and types of cells (foamy, Mallory bodies, etc.)
1979	London, UK	Peter Scheuer	Bile duct lesions
1980	Weiden, Germany	Gerhard Korb	Etiology of cholestasis and bile duct lesions
1981	Lisboa, Portugal	Amelia Baptista	Cholestatic syndromes
1982	Glasgow, UK	Roderick MacSween	Aspects of non-A–non-B chronic hepatitis
1983	Washington, DC, USA	Kamal Ishak	Aspects of hepatitis (including non-A–non-B)
1984	Meisterschwanden, Switzerland	Leonardo Bianchi	Types and patterns of liver necrosis
1985	Copenhagen, Denmark	Hemming Poulsen	Variants of piecemeal necrosis
1986	Leuven (Pellenberg), Belgium	Jan De Groote Valeer Desmet	Metabolic liver disease
1987	Saiger Höh (Titisee), Germany	Heribert Thaler	Metabolic liver disease
1988	Ittingen, Switzerland (in historic Kartause)	Martin Schmid	Hepatocellular carcinoma
1989	London, UK	Peter Scheuer	Hepatic epithelial tumors
1990	Weiden, Germany	Gerhard Korb	Liver in systemic disease: granulomas
1991	Graz, Austria	Helmut Denk	Hepatic granulomas
1992	Sesimbra, Portugal	Amelia Baptista	Liver pathology in transplantation (of the liver, kidney, bone marrow)
1993	Toronto, Canada	M. James Phillips	Transplantation pathology
1994	Rheinfelden, Germany	Fred Gudat Leonardo Bianchi	Grading and staging chronic hepatitis; primary sclerosing cholangitis and differential diagnosis
1995	Loch Lomond, UK	Roderick MacSween	Autoimmune cholangiopathies
1996	Leuven, Belgium	Valeer Desmet Jan De Groote	Autoimmune cholangiopathies
1997	Bethesda, USA	Kamal Ishak	Vascular diseases of the liver
1998	Sirmione (Brescia), Italy	Francesco Callea	Vascular diseases of the liver
1999	London, UK	Bernard Portmann	Lymphoproliferative diseases
2000	Newcastle upon Tyne, UK	Alastair Burt	Liver and immunosuppression
2001	Birmingham, UK	Stefan Hübscher	Hepatitis C infection in the immunocompromised host
2002	Ravello, Italy	Luigi Terracciano	Ductular reaction
2003	Leuven, Belgium	Tania Roskams Valeer Desmet	Ductular reaction
2004	Graz, Austria	Helmut Denk	Fatty liver diseases
2005	Rome, Italy	Francesco Callea	Fatty liver diseases
2006	Washington, DC, USA	Zachary Goodman	Hepatic fibrosis
2007	Lyon, France	Jean-Yves Scoazec	Hepatic fibrosis
2008	Cologne, Germany	Hans-Peter Dienes	Liver infections (excluding viral hepatitis)
2009	Halifax, Canada	Ian Wanless Eve Roberts	Tumors and tumor-like conditions
2010	Paris, France	Pierre Bedossa	Biliary mass lesions
2011	St. Louis, USA	Elizabeth Brunt	Cells of the sinusoid
2012	Mwanza, Tanzania	Francesco Callea	Pathology of sinusoids
2013	Noosa, Australia	Andrew Clouston	Well-differentiated hepatocellular lesions
2014	Ravello, Italy	Luigi Terracciano	Well-differentiated hepatocellular lesions
2015	Birmingham, UK	Stefan Hübscher	Acute hepatitis, including acute liver failure
2016	Adelaide, Australia	Alastair Burt	Patterns of acute liver injury
2017	Groningen, The Netherlands	Annette Gouw	Non-tumor vascular liver disease
2018	Athens, Greece	Dina Tiniakos	Drug-induced liver injury

## Gnomes’ scholarly contributions

One of the key missions of the Gnomes is to publish position/nomenclature/review articles to help advance the scientific understanding of liver disease [2]. To this end, there has been a regular production of Gnomes’ papers, totaling 12 at the time of the 50th year anniversary meeting in 2018 (Table 4). These papers have been widely cited, with the two most highly cited papers focusing on classification of hepatitis. The first Gnomes’ paper “A classification of chronic hepatitis” [5] has been cited 1016 times, while the most highly cited paper was published in 1995 and has 4747 citations: “Histological grading and staging of chronic hepatitis” [29].

**Table 4:** Papers of the Gnomes: “International Liver Pathology Group”

Number	Year	Title	Google Scholar Number of citations*	Web of Science Number of citations*
1	1968	A classification of chronic hepatitis [5]	1016	NA
2	1971	Morphological criteria in viral hepatitis [23]	117	NA
3	1974	Guidelines for diagnosis of therapeutic drug induced liver injury in liver biopsies [24]	45	NA
4	1977	Acute and chronic hepatitis revisited [7]	334	82
5	1981	Alcoholic liver disease: morphological manifestations [25]	123	68
6	1983	Histopathology of the intrahepatic biliary tree [26]	24	17
7	1988	The diagnostic significance of periportal hepatic necrosis and inflammation [27]	35	24
8	1994	Guidelines for the diagnosis and interpretation of hepatic granulomas [28]	57	27
9	1995	Histological grading and staging of chronic hepatitis [29]	4747	3393
10	2003	Histopathology of portal hypertension: a practical guideline [30]	55	35
11	2014	Pathology of the liver sinusoids [31]	39	23
12	2014	Well differentiated hepatocellular neoplasms of uncertain malignant potential: a proposal for a new diagnostic category [26]	37	28
Total		Total	6629	

*NA* not available

*As of March 29, 2020. Google Scholar citations include peer-reviewed articles as well as book chapters and other scholarly publications. The Web of Science includes almost exclusively primary articles and review articles

## Organizational structures that contribute to success

Fifty years is a long time for a small group to survive, let alone thrive—why has the Gnomes been successful and lasted so long? Peter Scheuer emphasized the deep satisfaction of sharing cases with other skilled pathologists, scientists, and clinicians who share a passion for liver disease [2], as did Valeer Desmet [4]. Professors Scheuer, Desmet, and Bianchi all highlighted the importance of openly sharing ideas, questions, and knowledge without fear of embarrassment [4, 7, 8]. In fact, Leonardo Bianchi explicitly noted that the egalitarian organization does not tolerate hierarchy in determining primacy of ideas during discussions [8] or in determining group direction (Figs. 1 and 2).

The notion of group fit comes through as a key element important to the health of the Gnomes [2, 4]. This makes sense because the social and intellectual fabric of small groups like the Gnomes can be easily torn if a member is disrespectful, does not fully participate, or is otherwise unable to integrate into the group.

A third key element noted by Valeer Desmet is the ability to organize the group’s efforts on a regular basis into meaningful contributions to the scientific literature [4]. All of the Gnomes are committed to academic endeavors, and it seems natural and entirely fitting that this aspect of Gnome membership would be highly valued.

Finally, the seamless integration of the Gnome partners and other family members into the social activities of the evenings and weekends brings to the Gnomes a true sense of community (Fig. 2).. Members get to know dimensions of each other not normally visible from typical academic meetings through shared social events, dinners, and other events. These informal environments promote trust, respect, and understanding.

## Central role of histomorphology

Leonardo Bianchi emphasized the importance of pre-meeting circulated slides and pre-meeting submission of diagnoses as a key element of the Gnomes [8]. Based on a single hematoxylin and eosin (H&E) slide, one unstained slide, and limited history, Gnomes members are asked to provide a diagnosis and limited differential. This unique approach requires each Gnome to commit to a diagnosis beforehand, allowing unbiased assessment for areas of consensus and areas of disagreement.

In addition, the careful examination of the H&E-stained slide at these meetings has led to the detection and interpretation of previously undescribed structural and cellular alterations. The Gnomes not only fully embrace modern approaches such as molecular pathology, but also understand the continued value of careful morphological studies. Examples of observations made at Gnomes meetings and subsequently described in follow-up studies include the strong association of calcification within alpha-1-antitrypsin globules with the Mmalton variant [25], the visualization of lipid droplets within eosinophilic inclusions of fibrinogen, corresponding to apo-beta-lipoproteins [32], and histological changes in fibrinogen storage disease of hypofibrinogenemia and hypo-apo-beta-lipoprotein [33].

The Gnomes’ slide sets are also very important as tools for educational training, including places or countries where H&E is the only available stain. In the last few years, some of the Gnomes have submitted scanned digital slides instead of glass slides, which also serve as an important educational tool, though the relative advantages and shortcomings of their use are still being explored.

At the end of the meeting, there also are opportunities for members to present their personal ongoing research. The opportunity to fully and openly discuss early ideas and data has been important in the process of refining many new ideas. There is a well-respected honor code that allows presentation of these initial ideas and early study results, without concern for other members absconding with them. These scientific topics incorporate and extend classical morphology using experimental models, molecular techniques, and biochemical methods, in order to better understand the basic principles of the disease and its morphological patterns. As one example, early work on the keratin nature of Mallory-Denk bodies was presented by Helmut Denk to the Gnomes, leading to vigorous and fruitful discussion by the group.

## Other examples of the small group model

The core elements of the Gnomes model have been replicated by another group of liver pathologists, founded in 1990 and called the *Elves*, assisted by Peter Scheuer, one of the founding Gnomes members. Their formal name “The International Liver Pathology Study Group” is easily confused with the formal name of the Gnomes (International Liver Pathology Group), so both groups generally use their more informal but distinctive names. The Elves have enjoyed great success and their history and accomplishments were recently reviewed [34].

## The future of the Gnomes

The Gnomes have consistently contributed to liver pathology for 50 years, but the future depends on the efforts of current members to keep it relevant, healthy, and productive. This depends on wise choices when selecting new members and on a rigorous and vigorous pursuit of the fundamental goal of the Gnomes: to improve the understanding of liver disease by tackling important issues in patterns of liver disease and in terminology.

For the first 50 years, the Gnomes focused their efforts on the histomorphologic patterns of disease. These activities remain important but now have to be more fully interwoven with the advances in the treatments for liver disease and improvements in non-invasive methods for diagnosing liver disease, assessing disease activity, assessing fibrosis, and integrating molecular findings into patient care. Starting about 10 years ago, the changes in treatment of liver disease have been rapid and sometimes stunning, with hepatitis C being a good example, in which rapid changes in the treatment and in non-invasive methods of assessing fibrosis have eliminated most of the clinical need for liver biopsy. These improvements in patient care are celebrated by all, especially Gnomes members.

Antipathy towards the value of liver pathology, however, is spreading among some clinicians, who express doubts about its usefulness in diagnosing and managing liver disease. The reasons for this are complex, but in part include the reduced exposure and understanding of liver pathology by newly trained physicians, who often receive considerably less training on normal histology and histopathology during medical school than formerly. In addition, there is a natural revision of diagnostic and treatment algorithms as new technology improves patient care. Refining the best fit for invasive and non-invasive methods in patient care takes time. Nonetheless, in all areas in which liver pathology can improve patient care, the Gnomes are committed to vigorously advancing the science of liver pathology interpretation.

The Gnomes experience indicates that small, agile professional groups can play an important role in medicine. These, and others like it, offer a number of specific benefits not available in larger specialty societies. We believe that nurturing such groups advances science and medicine in important, meaningful ways. The documentation of the Gnomes approach can provide a road map for the formation of future groups with a specific scientific focus.

## Electronic supplementary material

ESM 1 (DOCX 20 kb)
