# Supplementary material for: Fifty years of impact on liver pathology: a history of the Gnomes
Source: Virchows Arch. 2020 Jun 30;478(2):191–200. doi: 10.1007/s00428-020-02879-5 (PMC7969554; doi:10.1007/s00428-020-02879-5)
Supplement: Supplementary file 1 — (DOCX 20 kb) [file 428_2020_2879_MOESM1_ESM.docx]

**Supplementary material**

**Appendix. Customs of the Gnomes**

1. The meeting location rotates each year, with members taking turns hosting the meetings
2. The theme/topic is chosen by the upcoming host, in discussion with the entire group, and announced at the year’s prior meeting.
3. If there is sufficient interest, topics are discussed at two consecutive meetings
4. Cases from each member are sent to every other member of the group before the meeting. There are two cases (sometimes three) that are circulated, which consist of limited history, one H&E and, when possible, one unstained section. Glass slides are preferred but digital images can also suffice. Cases should be “on theme” as much as possible, but can take the theme into new and interesting directions
5. Each member independently reviews the pre-meeting circulated slides and sends in their personal diagnosis and comments to the meeting host before the meeting
6. The host collates the diagnoses from each member and puts together a meeting binder with the diagnoses. A binder is given to each Gnome at the beginning of the meeting.
7. The meeting lasts three days: (with welcome reception on Wednesday night), Thursday, Friday, Saturday, with departures on Saturday evening or Sunday. The early meetings lasted two days, but this was soon expanded to give sufficient time for discussion. [2]
8. There is a program for persons accompanying Gnomes members during the meeting hours, that includes sightseeing, educational activities, and time for relaxation. Accompanying members join members for evening dinners and social events. In many ways, these social interactions are the heart of the Gnomes meetings.
9. At the meeting, each Gnome member presents their case, usually in alphabetic order by city of origin (but the host can choose to proceed in any order they want), and discusses their case with the group
10. Gnomes are free to comment and discuss at will, openly, frankly, and civilly. This discussion of cases in many ways is the soul of the meeting.
11. Gnomes who are interested can also present some of their personal research in sessions held at the end of the meeting
12. Younger colleagues of the host are free to attend the academic portion of the meeting and observe, though they do not actively participate.[2]
13. A business meeting is held at the end of the meeting on Saturday morning. The business meeting generally follows this format:
    1. The host or their designee summarizes the results of the meetings discussion, highlighting new insights, points of consensus, and points of disagreement.
    2. The potential of the meeting’s presentations and discussions to serve as the basis for a useful review article or position statement is discussed.
       1. If there appears to be enough material that is worthy of publication, then a writing committee is formed, usually chaired by the host.
       2. The writing committee prepares a draft and shepherds the manuscript through to publication. All Gnomes attending the meeting are coauthors.
    3. Member status for current members is discussed as needed (pending retirements, members otherwise unable to fully participate by circulating slides and attending) and consideration, when needed, of names for possible new members.
    4. Next year’s location is agreed upon (and often locations for the next several years thereafter). This process is flexible, with members volunteering to host in their approximate order of “not having done it for a while”, but with accommodation for those who want to host a meeting more quickly than order would suggest, for personal reasons.
    5. Next year’s topic is chosen by next year’s host, in discussion with the entire group.
14. The final Gnomes dinner on Saturday night holds the only real (but very simple) ceremony:
    1. The Gnome who has been a member the longest formally thanks the host on behalf of the Gnomes.
    2. One of the Accompanying Gnomes formally thanks the host and those that organized the Gnome-mates program.
    3. The host places on the head of the next year’s host the official red hat of the Gnomes. This hat was introduced in 1990 by Peter Scheuer and is held with special reverence (and fear of losing) by the upcoming year’s host until the meeting is concluded, and then the hat is passed on to the next host.

15. The induction of new members is as follows

a. They are invited personally by the host of the meeting that will be their first meeting. The host explains the process.

b. They participate in the pre-meeting slide circulation (also submitting cases for circulation), submit their diagnoses to the host before the meeting, present their cases at the meeting, and participate in all academic and social aspects of the meeting.

c. The candidate excuses themselves for a portion of the business meeting, during which time the members of the Gnomes discuss the “fit” of the candidate in regard to the four key elements of a Gnome member: scientific interest, diagnostic expertise, collegiality, and friendship. After a full and frank discussion, the Gnomes members vote on membership.

- 1. When membership is extended, the obligations of membership are fully articulated: (1) meetings should not be missed other than for true emergencies or illness; (2) members should respond reasonably promptly to host emails; (3) slides should be circulated on time and diagnoses submitted to the host on time; (4) full and civil participation in the academic and social program is anticipated; (5) if circumstances change and a Gnome is no longer able to meet these requirements, then the Gnomes resigns/retires, to an emeritus status when in good standing.
